# Nutraceutical Properties of Mulberries Grown in Southern Italy (Apulia)

**DOI:** 10.3390/antiox8070223

**Published:** 2019-07-16

**Authors:** Carmine Negro, Alessio Aprile, Luigi De Bellis, Antonio Miceli

**Affiliations:** Department of Biological and Environmental Sciences and Technologies (DiSTeBA), Salento University, Via Prov. le Lecce-Monteroni, 73100 Lecce, Italy

**Keywords:** mulberry (*Morus nigra*; *Morus alba*), simple sugars, organic acids, phenol compounds, high performance liquid chromatography/diode array detector/mass spectrometry anthocyanins, antioxidant activity, anti-inflammatory activity

## Abstract

In this work, for the first time, were analyzed mulberry genotypes grown in Apulia (Southern Italy, Salento region) were analyzed. Two local varieties of *Morus alba* (*cv. Legittimo nero* and *cv. Nello*) and one of *Morus nigra* were characterized for content in simple sugars, organic acids, phenols, anthocyanins; fruit antioxidant activity (AA) was also evaluated by three different methods (2,2-Diphenyl-1-picrylhydrazyl, DPPH; 2,2′-Azino-bis(3-ethylbenzothiazoline-6-sulfonic acid), ABTS; and Ferric reducing antioxidant potential, FRAP test). The results showed that the sugars amount ranged between 6.29 and 7.66 g/100 g fresh weight (FW) while the malic and citric acids content was low, at about 0.1–1 g/100 g FW. Mulberries are a good source of phenols which are present in higher values in *M. nigra* and *M. alba cv. Legittimo nero* (485 and 424 mg Gallic Acid Equivalent (GAE)/ 100 g FW, respectively). The high performance liquid chromatography/diode array detector/mass spectrometry (HPLC/DAD/MS) analysis identified 5 main anthocyanin compounds present in different concentrations in each variety of mulberry: cyanidin 3-sophoroside, cyanidin 3-glucoside, cyanidin 3-rutinoside, pelargonidin 3-glucoside, pelargonidin 3-rutinoside. The highest concentration of anthocyanins was determined in *Morus alba Legittimo* (about 300 mg/100 g FW) while the lowest content (about 25 mg/100 g FW) was measured in *M. alba cv. Nello. Morus nigra* showed a good AA in comparison with the different *M. alba* genotypes with all the used methods; its AA was equal to 33, 26 and 21 μmols Trolox/g FW when using DPPH, ABTS and FRAP tests, respectively. All genotypes showed an anti-inflammatory activity (measured by cyclooxygenase (COX) inhibitory assay) which was also compared with two commercial anti-inflammatory drugs. The data obtained support the high biological qualities of mulberry fruits and their diffusion in human nutrition.

## 1. Introduction

The mulberry is a dicotyledon plant belonging to the genus *Morus* of the Moraceae family and generally it is well known in sericulture and silk industry, since it is the only food plant for the domesticated silkworm *Bombyx mori* [1]. There are 24 species of *Morus* with at least 100 known varieties; it is widely distributed in Asia, Europe, North and South America, and Africa [2]. The most important species are *Morus alba*, with fruit colors ranging from white to dark red, and *Morus nigra* with dark red fruits mainly; both species have excellent productions in Mediterranean climate areas [3].

Almost all the parts of the mulberry-tree are used for their pharmacological actions. The leaves have been shown to have diuretic, hypoglycemic, and hypotensive activities [4,5], whereas the root bark has long been used for anti-inflammatory, antitussive, and antipyretic purposes [5]. Different studies underlined that these properties are due to hydroxylated alkaloids, flavonoids and Diels-Alder type adducts which seem to be responsible for a high α-glucosidase inhibition [6,7,8], improving the glucose levels in patients affected by type II diabetes [9,10].

Moreover, the mulberry fruit has numerous biologically active compounds like phenols, flavonoids, anthocyanins, carotenoids, essential fatty acids, ascorbic acid, and various organic acids [11,12], resulting in high antioxidant activities, especially in fully ripened fruits [10]. Mulberries can be consumed both fresh and processed such as syrup, jam, pulp, ice-cream, etc. Traditionally they are used as a warming agent, as a preparation against dysentery and as a tonic, sedative, laxative, odontalgic, anthelmintic, expectorant, and emetic. Moreover, a great deal of evidence suggests their potential role in the prevention of cancers, cardiovascular diseases, apoptosis, neurotoxicity and neurodegeneration [10,13] and oxidative stress. Several studies suggest that the reactive oxygen species (ROS) and reactive nitrogen species (RNS) play important negative roles by oxidizing DNA and other molecules, leading to age-related diseases [14,15,16]. In particular the brain, which has a fundamental role in cognitive dysfunction usually associated with neuro-degenerative problems, is the most sensitive organ to the oxidative stress because of its high oxygen need, high metabolic rate and relative low antioxidative defense mechanisms [17,18,19]. In addition, recently, higher antinociceptive properties have been reported for black mulberry fruits, which inhibited the expression of inflammation-related proteins [20].

These biological activities are correlated to their polyphenol components, in particular anthocyanins, a large group of water-soluble pigments responsible for colors (orange, red and blue) of flowers, fruits and vegetables and principally known for the high antioxidant activity, anti-inflammatory potential and reduction of liver injury (chemo-and hepatic protective role) [21,22]. Cyanidin 3-*O*-glucoside (C3G), cyanidin 3-*O*-rutinoside (C3R), pelargonidin 3-*O*-glucoside (P3G), pelargonidin 3-*O*-rutinoside (P3R) are the main anthocyanins identified in mulberries and their antioxidant power is very high [21,23].

Mulberry grows in a wide range of climatic, topographical, and soil conditions which can affect the chemical composition and nutritional status of the fruits. Although mulberry fruits have been characterized in different parts of the world, information on fruits from plants grown in Southern Italy (Salento, South Apulia) has not been obtained. In the present study, therefore, fruits of local variety of *Morus alba* and *Morus nigra* have been characterized for their nutritional components by evaluating the content of simple sugars, organic acids, total phenolic compounds, *o*-diphenolic compounds, anthocyanins, and antioxidant and anti-inflammatory properties.

## 2. Materials and Methods

### 2.1. Chemicals

2,2-Diphenyl-1-picrylhydrazyl (DPPH), 2,2′-Azino-bis(3-ethylbenzothiazoline-6-sulfonic acid) diammonium salt (ABTS), Gallic acid, Folin-Ciocalteu reagent, Trolox (6-hydroxy-2,5,7,8-tetramethylchroman-2-carboxylic acid), acetonitrile, acetone, methanol and water HPLC grade were purchased from Sigma-Aldrich Chemical Co. (St. Louis, MO) and they are of analytical grade. Cyanidin-3-O-Glucoside analytical grade was purchased from Extrasyntese (Genay, France), and anti-inflammatory Kit was purchased from Cayman Chemicals cat. Number 560131 (Ann Abor, MI, USA). Sugar kit and organic acids kit were purchased from R-Biopharm Italia Srl, cat. Numbers 10716260035, 10139076035, 10139068035 (Cerro al Lambro, Milano, Italy).

### 2.2. Extraction and Purification Juice

The analytical determinations were carried out on one local variety of *Morus nigra* and on two varieties of *Morus alba* known as *Morus alba cv Legittimo nero* and *Morus alba cv Nello*, which were harvested at full maturity in the Corigliano town fields (40°9′38”,16 N, 18°15′36”,72 E) (Province of Lecce) during June–July 2015 and stored at −20°C prior to the analysis.

Juice was obtained by centrifugation and used to quantify the sugars (sucrose, fructose and glucose) and citric and malic acid amounts by an enzymatic spectrophotometric kit provided by R-Biopharm Italia (see “Chemicals” paragraph 2.1) and expressing the results as g/100 g of fresh weight (FW) and, after filtration with Watmann n. 1, for determination of antioxidant activity. The moisture was determined in accordance to the AOAC method [24].

For the determination of phenolic compounds, a raw extract of each mulberry sample was obtained homogenizing 25 g of fresh plant material (3 min. at 10,000 rpm with Ultraturrax) using 100 mL of cold acetone 70% (*v*/*v*) acidified with 0.1% HCl. The homogenate was stirred for two hours in the dark at 4 °C, then centrifuged at 5000× *g* for 10 min. On the pellet, two further extractions were carried out in the same way. The supernatants were dried at reduced pressure and re-suspended with ultrapure water (HPLC grade) acidified with 0.1% HCl, thus obtaining a final volume of 100 mL (1:4 *w*/*v*).

### 2.3. Total Phenolics and Anthocyanins Determination

Total phenolics compounds (TPC) were measured by the Folin Ciocalteau spectrophotometric method using gallic acid as a standard and expressing the results as mg Gallic Acid Equivalent (GAE)/100 g FW [25]. Moreover, *o*-diphenolics compounds (ODC) were determined by the Arnow spectrophotometric method and expressing the results as mg chlorogenic acid/100 g FW [26].

In order to evaluate the anthocyanin amount using high performance liquid chromatography/diode array detector/mass spectrometry (HPLC/DAD/MS), the mulberry raw extracts were purified by solid phase extraction (SPE) using cartridge Strata X (Phenomenex Italia, Castel Maggiore, Bologna, Italy). After activation of the SPE cartridge with 2 mL pure methanol and 5 mL bi-distilled water, 20 mL raw extract were loaded and washed with 10 mL acidified bi-distilled water and 5 mL ethyl acetate to remove sugars and less polar flavons respectively; finally, 20 mL acid methanol were used to recover the anthocyanins. The purified extract was dried under vacuum and re-suspended with HPLC water acidified with 0.1 % HCl.

The identification was completed using an HPLC Agilent 1100 system (Agilent Tecnnologies, palo Alto, CA, USA) coupled with an Agilent DAD sensor (detection wavelength 520 nm) and Agilent ESI/MS spectrometer 6100 in positive ionization mode as reported by Negro et al. [27]. The separation was carried out at 30 °C with a gradient elution program at a flow rate of 0.8 mL/min using a Phenomenex Gemini C18 250 × 4.6 mm, 5 µm separation column. The mobile phases consisted of water plus 2% formic acid (A) and water:formic acid:acetonitrile 48:2:50 (B). The following multistep linear gradient was applied: 0 min, 6% B; 15 min, 30% B; 25 min, 50% B; 30 min, 60% B. The injection volume in the HPLC system was 20 μL. The quantification of anthocyanin was achieved using calibration curves of the authentic chemical standards cyanidin 3-glucoside (C3G) and moreover, total anthocyanins (TA) was determined as being the sum of the area of the single compounds.

### 2.4. Antioxidant Tests

The antioxidant activity tests were performed by spectrophotometric assays using three different methods: DPPH, ABTS and FRAP (Ferric Reducing Antioxidant Power) test using a 96-well microplate according to Oki et al. [10], Re et al. [28] and Benzie and Strain [29], respectively. In all the assays, Trolox was used as a standard and results are expressed in terms of microgram of Trolox Equivalent Antioxidant Capacity (TEAC) per g of FW of sample.

### 2.5. Anti-Inflammatory Test

Anti-inflammatory activity (AI) was evaluated determining the cyclooxygenase activity (COX). The COX-1 and COX-2 inhibitory assay was carried out using COX Inhibitor Screening Assay Kit (Catalogue N° 560131, Cayman Chemicals, Ann Abor, MI, USA) according to the instructions provided by the manufacturer and as previously reported [27]. The AI was determined using the phenolic extract and results are expressed as inhibitory activity (IC_50_, µg/mL).

### 2.6. Statistical Analysis

Data are reported as the mean ± SD and three biological replicates are carried out for each sample. Statistical evaluation was conducted by ANOVA, followed by multicomponent Duncan’s test (*p* < 0.05) to discriminate among the mean values.

## 3. Results and Discussion

### 3.1. Simple Sugars, Organic Acids and Total Phenolics Content

The moisture and quantity of simple sugars, malic and citric acids, TPC and ODC were reported in Table 1. The moisture content in the fruits is very close, about 78%, according to the literature [1,11]. Sucrose was completely absent in all the samples analyzed; *M. nigra* had 7.66 g/100 g FW of sugars whereas the glucose and fructose contents were similar and equal to 3.94 and 3.72 g/100 g FW, respectively.

The fruits of other varieties showed a similar trend and although the glucose and fructose were equivalent, the total amount of sugars was lower corresponding to about 6 g/100 g FW. These values are in agreement as reported for the species of mulberry (*M. nigra*, *M. alba* and *M. laevigata*) cultivated in Pakistan [1], but they are notably inferior when compared to *M. nigra*, *M. alba* and *M. rubra* grown in Turkey for which the content of glucose and fructose was corresponding to about 6–7 g/100 g FW, respectively [30]. The organic acid content (malic and citric) was low and ranged between 0.13 and 1.02 g/100 g FW for *M. alba cv Nello* and *M. nigra*; *M. nigra* showed a greater quantity of citric acid of 0.92 g/100 g FW and this was in accordance with what was found in different varieties of Turkish mulberries where the amount of citric acid ranged between 0.39 and 1.08 g/100 g FW [30].

The differences between species in terms of citric and malic acid content might be caused by genetic factors as well as agronomic practices and ecological factors (temperature, soil conditions, humidity, ect.). In fact, Koyuncu [31] reported a variable amount of citric and malic acid that ranged between 0.5 to 2.3 g/100 g FW and 3.5 to 19.8 g/100 g FW for different genotypes of mulberry fruits cultivated in Turkey. These differences are also evident in fruits grown in different localities of the same country; Koyuncu [31] reported different amounts in citric and malic acid for black mulberry fruits from two locations ranged between 0.8 to 1.3 g/100 g FW and 5.7 to 9.9 g/100 g FW, respectively. In other Turkish mulberry genotypes, the content of malic and citric acid was very different, varying from 12.9 to 21.8 g/100 g FW and from 2.1 to 4.1 g/100 g FW, respectively [11].

Organic acids are water soluble and together with the sugars contribute to the taste of vegetables and fruits. The ratio of the total acid amount to the content of sugars in fruits is a criterion for the maturity; moreover, organic acids have a high impact on taste because of their conditioning (a reduction) on sweetness and their favoring effect on sourness. In addition, the type and the amount of acidity could be used for food decay; if the fruit is molded during the wait, there is an increment of some organic acids which seems also to have a significant impact on the purity control [30].

The amount of TPC and ODC, evaluated as mg of gallic acid equivalent (GAE) and mg of chlorogenic acid, respectively, is also reported in Table 1. The data showed that in *M. nigra* and *M. alba Legittimo* the content of TPC was equal to about 485 and 423 mg GAE/100 g FW, respectively and corresponding to more than three times the TPC present in *M. alba* cv *Nello*. *M. nigra* showed an ODC content of 102.21 mg/100 g FW, an amount about 1.8 and 4 times higher than that present in *M. alba* cv *Legittimo nero* and *M. alba* cv. *Nello*, respectively.

Mulberry fruits are a good source of phenolic compounds and the results clearly showed that fruit analyzed had high total phenolic content, nevertheless they showed wide differences in comparison to the literature data. In fact, TPC in mulberry fruits was reported which ranged from 104.8 to 213.5 GAE mg/100 g FW for eight Thai genotypes [32], from 158.4 to 249 mg/GAE 100 g FW and from 100.5 to 348.8 mg GAE/100 g FW, respectively for mulberries harvested in different Turkish sites [33,34], and from 76.7 to 180 mg GAE/100 g FW in fruits grown in Spain [35]. However, data reported by Imran et al. [1] and by Ercisli et al. [12] showed that the TPC content in fruits collected in the northern region of Pakistan and in North-East Anatolia (Turkey) was very high, ranging from 880 to 1650 and from 1943 to 2237 mg GAE/100 g FW, respectively. This great variability in the content of total phenols is related to the genotype, the conditions of growth and cultivation [22] and could be influenced by fruit moisture, too. It is known, in fact, that the plant can accumulate phenolic compounds under various stress conditions (heat, UV light, pathogen attack, etc.); in particular, climatic changes like low or high-temperature stress can promote the production of the phenolic compounds [36,37,38].

### 3.2. Anthocyanins Analysis

**Figure 1:**
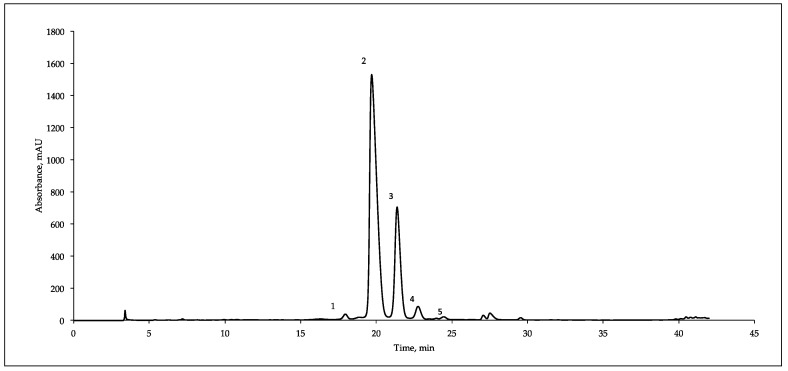
Typical HPLC/DAD/MS analysis (recorded at 520 nm) of the anthocyanins present in mulberry fruit extracts (*M. nigra*). (1) cyanidin-3-*O*-sophoroside, (2) cyanidin-3-*O*-glucoside, (3) cyanidin-3-*O*-rutinoside, (4) pelargonidin -3-*O*-glucoside and (5) pelargonidin -3-*O*-rutinoside.

Figure 1 reports a typical HPLC/DAD/MS separation of the anthocyanins present in mulberry fruits. Five components were identified on the basis of the UV and MS spectra and retention time, corresponding to cyanidin-3-O-sophoroside (C3S, m/z 611, peak 1), cyanidin-3-O-glucoside (C3G, m/z 449, peak 2), cyanidin-3-O-rutinoside (C3R, m/z 595, peak 3), pelargonidin-3-O-glucoside (P3G, m/z 433, peak 4) and pelargonidin-3-O-rutinoside (P3R, m/z 579, peak 5). Our results are in agreement with the data reported some years ago by Dugo et al. [39] that identified a mixture of five different anthocyanidin glycosides in Italian mulberries cultivars but are discordant with Pawloska et al. [13], which revealed four anthocyanins in *M. nigra* fruits harvested in Benevento (South Italy).

The anthocyanins identified and the total anthocyanin content (TA), calculated as the sum of the single compounds, were shown in Table 2. The TA in *M. alba Nello* was 24.7 mg/100 g FW, was about 8 and 12 times lower than that determined in *M. nigra* and *M. alba Legittimo nero*, corresponding to 206 and 289 mg/100 g FW, respectively. According to earlier reports, TA content was 2–30, 0.3–83, 1.5–615 mg/100 g FW respectively for genotypes grown in Turkey, Korea and India [34,40,41]. C3G and C3R are the anthocyanins most represented with amounts ranging from 18.2 to 212 and from 5.2 to 71.8 mg/100 g FW, respectively. P3G ranged from 1.6% to 5% of the TA content while C3S was sligtly higher in *M. nigra*, representing about 1.5% of the TA in comparison to 0.17% and 0.56% for the other genotypes.

### 3.3. Antioxidant Activity

Table 3 reports the mulberry AA measured by three different tests. Data indicate clearly that *M. nigra* showed the highest antioxidant activity corresponding to about 33, 26 and 21 μmol Trolox/g FW, respectively by the DPPH, ABTS and FRAP tests.

In the DPPH scavenging activity test, all the mulberry genotypes showed an AA significantly higher than ABTS and FRAP test. In fact, *M. alba Legittimo nero* exhibited an antioxidant capacity that was about two times greater than that measured with the other methods and *M. alba Nello* showed a capacity that was about 10 times lower when assessed by the FRAP test.

These data could be the result of a different qualitative and quantitative composition of phenolic compounds of the three mulberries. Further studies are needed to prove this. Moreover, we cannot exclude the possibility that the quenching mechanisms of the different compounds are more efficient against DPPH• than ABTS•^+^.

However, the observed antioxidant activity is probably due to the set of phenolic compounds present in the juice; in addition to the anthocyanin effect, it is known that there are significant quantities of other phenolic compounds such as gallic and cinnamic acid, procyanidin B1, catechin and quercetin [32].

Moreover, these results are in agreement with the literature data, in particular with the work of Ercisli et al. [33] that reports for *M. nigra* and *M. rubra* an AA ranging between 16 and 21.2 μmol Trolox/g FW and between 9.2 and 12.1 μmol Trolox/g FW when it was estimated by DPPH; instead, the range was between 12.3 to 14.1 μmol Trolox/g FW and from 4.9 to 8.1 μmol Trolox/g FW, respectively for *M. nigra* and *M. rubra* when measured by the FRAP test [33]. Also, the radical scavenging activity measured using ABTS system was in agreement with the values reported for different mulberry genotypes (black and/or red mulberries) grown in various Turkish regions, ranging from 6.8 to 14.4 μmol Trolox/g FW and from 5.1 to 7.3 μmol Trolox/g FW, respectively, in *M. nigra* and *M. rubra* [34].

### 3.4. Anti-Inflammatory Activity (AI)

The anti-inflammatory activity is related to the ability of some compounds to inhibit the two isoforms of the cyclooxygenase enzyme, COX-1 and COX-2; this activity was demonstrated for the anthocyanins isolated from raspberries and sweet cherries (cyanidin-3-glucosyl-rutinoside and cyanidin-3-rutinoside [42] and also for the anthocyanidins as cyanidin and malvidin [43].

The results of the AI in vitro assay are reported in Table 4; data indicate for all the extracts a relevant biological activity. The IC_50_ values relative to COX-1 ranged between 125 and 185 µg/mL TA and from 64 and 97 µg/mL TA relative to COX-2 for *M. nigra* and *M. alba Nello* genotypes, respectively. Employing identical analysis conditions, the AI of ibuprofen and nimesulide (positive controls, two synthetic anti-inflammatories compounds), were equal to 6 and 5 µg/mL, respectively, for COX-1 assay, and to 5 and 2 µg/mL for COX-2 assay. To our knowledge, it is the first time that mulberry extracts AI was determined in vitro using the ELISA methodology. Several authors have evaluated pure anthocyanins or different fruit extracts (such as cherry, blueberry, blackberry, etc) employing different assay methods showing that among aglycones, cyanidin was particularly active in the COX-2 assay [39]. Comparing IC_50_ values for different fruits from literature data, for pomegranate values were reported relative to COX-1 between 249 and 145 µg/mL and from 175 and 75 relative to COX-2, respectively for different genotypes grown in Salento area (Southern Apulia) [27]; the cherries showed values up to 130 µg/mL whereas blueberries, which are known to have high AI, had a range of 400–800 µg/mL depending to the ecotypes [42].

## 4. Conclusions

To our knowledge, this is the first time that mulberry genotypes growing in the Salento area have been characterized, particularly in terms of phenols, anthocyanin analysis and biological properties. The biochemical analysis of different mulberry genotypes confirmed that this fruit is rich in biologically active substances such as total phenols, o-diphenols, and anthocyanins, which are also responsible for strong antioxidant and anti-inflammatory properties. Considering the high concentration of total phenols, particularly anthocyanins, and the free anti-scavenging properties, this “minor” fruit can be considered an excellent source of antioxidant compounds and *M. nigra* and *M. alba Legittimo nero* seem to be the most interesting genotypes. Moreover, in relation to the sugars and organic acid contents, it is important to observe that their contents positively influence consumer opinions.

Mulberry should be classified as a “functional food” and its use as fruit, juice, jams, or as a muesli component may be recommended in human nutrition because of its biological qualities. In fact, the antioxidant and anti-inflammatory activities of the mulberry suggest that its diffusion and consumption would be beneficial to human health; hopefully, this fruit, if included in a regular diet, might be able to alleviate and/or control different pathologies.

Moreover, the mulberry may represent a good plant species for more extensive re-implantation in marginal zones and/or degraded lands. Its cultivation in a territory such as the Salento, a site strongly suffering from a devastating *Xylella fastidiosa* infection, might give a positive boost to agriculture and, at the same time, protect the environment from further degradation.

## Figures and Tables

**Table 1:** Amounts of moisture (%), glucose, fructose, malic and citric acid (expressed as g/100 g FW), total phenolic compounds (TPC) (mg Gallic Acid Equivalent (GAE)/100 g FW) and *o*-diphenolic compounds (ODC) (mg of chlorogenic acid /100 g FW) in different mulberry fruit varieties. Values represent the results of three determinations ± SD; means with different letters in the same column are significantly different from each other (*p* < 0.05) according to the multicomponent Duncan’s test.

Genotype	Moisture	Glucose	Fructose	Malic Acid	Citric Acid	TPC	ODC
*M. nigra*	78.2 ± 1.1 ^a^	3.9 ± 0.1 ^a^	3.7 ± 0.1 ^a^	0.1 ± 0.1 ^a^	0.9 ± 0.1 ^a^	485.5 ± 7.1 ^a^	101.2 ± 6.2 ^a^
*M. alba Legittimo nero*	77.7 ± 1.3 ^a^	3.3 ± 0.1 ^a^	3.0 ± 0.1 ^b^	0.1 ± 0.1 ^a^	0.2 ± 0.1 ^b^	423.6 ± 4.2 ^b^	60.4 ± 3.1 ^b^
*M. alba Nello*	77.6 ± 1.2 ^a^	3.2 ± 0.1 ^a^	3.2 ± 0.1 ^b^	0.1 ± 0.1 ^a^	0.1 ± 0.1 ^b^	141.2 ± 6.1 ^c^	26.2 ± 2.1 ^c^

**Table 2:** Amounts of cyanidin-3-*O*-sophoroside (C3S), cyanidin-3-*O*-glucoside (C3G), cyanidin-3-*O*-rutinoside (C3R), pelargonidin-3-*O*-glucoside (P3G) and pelargonidin-3-*O*-rutinoside (P3R) and Total anthocyanins (TA) in mulberry fruits. Values are expressed as C3G mg/100 g FW and represent the results of three determinations ± SD. Means with different letters in the same column are significantly different from each other (*p* < 0.05) according to the multicomponent Duncan’s test.

Genotype	C3S	C3G	C3R	P3G	P3R	TA
*M. nigra*	3.1 ± 0.3 ^a^	138.6 ± 1.5 ^b^	52.3 ± 2.1 ^b^	10.4 ± 1.1 ^a^	1.7 ± 0.1 ^a^	206.1 ± 1.8 ^b^
*M. alba Legittimo nero*	0.5 ± 0.1 ^b^	212.2 ± 1.1 ^a^	71.8 ± 0.3 ^a^	4.7 ± 0.2 ^b^	0.1 ± 0.1 ^c^	289.2 ± 0.9 ^a^
*M. alba Nello*	0.1 ± 0.1 ^c^	18.2 ± 0.4 ^c^	5.2 ± 0.2 ^c^	1.0 ± 0.1 ^c^	0.2 ± 0.1 ^b^	24.7 ± 0.3 ^c^

**Table 3:** Antioxidant activity of different mulberry genotypes evaluated by DPPH, ABTS and FRAP test. Results are expressed as μmol Trolox Equivalents/g FW. Values represent the results of three determinations ± SD; means with different letters in the same column are significantly different from each other (*p* < 0.05) according to the multicomponent Duncan’s test.

Genotype	DPPH Test	ABTS Test	FRAP Test
*M. nigra*	32.9 ± 0.7 ^a^	26.1 ± 1.5 ^a^	21.3 ± 1.1 ^a^
*M. alba Legittimo nero*	22.7 ± 1.4 ^b^	11.6 ± 2.3 ^b^	10.6 ± 1.5 ^b^
*M. alba Nello*	18.5 ± 2.3 ^c^	7.3 ± 2.1 ^b^	1.7 ± 0.9 ^c^

**Table 4:** Anti-inflammatory activity (AI) of mulberry genotypes measured as COX-1 and COX-2 inhibitory activity expressed as IC_50_, µg/mL extract. Values represent the results of three determinations ± SD; means with different letters in the same column are significantly different from each other (*p* < 0.05) according to the multicomponent Duncan’s test.

Genotype/Component	AI (IC_50_, µg/mL)	
	COX1	COX2
*M. nigra*	125 ± 5 ^b^	64 ± 7 ^b^
*M. alba Legittimo nero*	140 ± 7 ^c^	81 ± 8 ^c^
*M. alba Nello*	185 ± 9 ^d^	97 ± 5 ^c^
Nimesulide	5 ± 1 ^a^	2 ± 1 ^a^
Ibuprofen	6 ± 1 ^a^	5 ± 1 ^a^
